# Subclinical Atherosclerosis in Primary Sjögren's Syndrome: Does Inflammation Matter?

**DOI:** 10.3389/fimmu.2019.00817

**Published:** 2019-04-17

**Authors:** Elena Bartoloni, Alessia Alunno, Giacomo Cafaro, Valentina Valentini, Onelia Bistoni, Angelo Francesco Bonifacio, Roberto Gerli

**Affiliations:** Rheumatology Unit, Department of Medicine, University of Perugia, Perugia, Italy

**Keywords:** Sjögren's syndrome, atherosclerosis, inflammation, endothelium, cardiovascular

## Abstract

Sjögren's syndrome (SS) is a systemic autoimmune disease mainly characterized by inflammatory involvement of exocrine gland. Atherosclerosis is a complex process leading to plaque formation in arterial wall with subsequent cardiovascular (CV) events. Recently, numerous studies demonstrated that SS patients bear an increased CV risk. Since activation of immune system is a key element in atherosclerosis, it is interesting to analyze whether and how the autoimmune and inflammatory events characterizing SS pathogenesis directly or indirectly contribute to atherosclerosis risk in these patients. An increase in circulating endothelial microparticles and integrins, which may be a consequence of endothelial damage and impaired repair mechanisms, has been demonstrated in SS. Increased endothelial expression of adhesion molecules with subsequent infiltration of inflammatory cells into arterial wall is also a critical event in atherosclerosis. The early inflammatory events taking place in the atherosclerotic plaque cause an increase in alarmins, such as S100A8/A9, which seems to be associated with SS disease activity and, in turn, induce up-regulation of interleukin (IL)-1β and other pro-atherogenic cytokines. Interestingly, increased IL-1β levels were also detected in tertiary lymphoid structures developing in vessel adventitia adjacent to the atherosclerotic plaque, suggesting a direct role of IL-1β in this process. Similar to these structures, germinal center-like structures arising in SS exocrine glands are also tertiary lymphoid systems where T-helper (Th) cell subsets govern the adaptive immune response. Th1 cells are the most prevalent subtype and have been shown to be strongly involved in both SS pathogenesis and atherosclerosis. Th17 cells are attracting great interest and few studies showed its importance in SS development. Albeit in low amounts, a Th17 signature was also detected in atherosclerotic plaques and some animal models demonstrated a significant pro-atherogenic role and positive effects of IL-17A blockade. Despite the fact that T cells have a pivotal role in the inflammatory process that ultimately leads to atherosclerosis, B cells have also been detected in atherosclerotic plaques, although their exact role is still mostly unknown with studies showing contrasting results. In this scenario, the role of inflammation in atherosclerosis pathogenesis in patients with SS needs to be further explored.

## Introduction

Inflammation plays a pivotal role in the pathogenesis of atherosclerotic vascular damage and the causal relationship between chronic inflammation an atherosclerotic cardiovascular (CV) disease has been widely explored in the last years. It is now accepted that atherosclerotic endothelial damage is the result of a complex and still unknown interplay between multiple pathogenic players, including traditional CV risk factors, immune-mediated mechanisms, and inflammatory triggers (1). Early in the course of atherosclerosis, activation of vascular smooth muscle cells and upregulation of pro-inflammatory mediators, including cytokines, vascular endothelial growth factors, and leukocyte adhesion molecules, represent the first step of atherosclerotic vascular damage. In this setting, it is intriguing to note that the same inflammatory markers involved in the pathogenesis of arterial wall atherosclerotic damage have been demonstrated to independently predict CV disease events (2). Interleukin (IL)-6 and high-sensitive C reactive protein (hsCRP) have been shown to be independent predictors of CV disease and ongoing research is directed to test the hypothesis that targeting chronic inflammation could prevent atherosclerosis progression and reduce CV events (3).

Of consequence, the evidence that the same pro-inflammatory molecules driving atherosclerosis play an essential role in the pathogenesis of systemic autoimmune rheumatic diseases (ARDs) discloses intriguing scenarios. Increased rate of CV events as well as enhanced prevalence of subclinical atherosclerosis, documented by imaging and vascular function instrumental methods are well-recognized in patients with ARDs, including systemic lupus erythematosus (SLE), rheumatoid arthritis (RA), systemic sclerosis (SSc), and Sjögren's syndrome (SS) (4). In these patients, accelerated subclinical atherosclerosis has been associated not only to increased prevalence of classical CV risk factors, which induce atherosclerotic arterial wall damage enhancing the risk of CV disease (5, 6), but also to chronic inflammatory burden (7). Decisive evidence supports that, in particular in SLE and RA patients, long-standing systemic inflammation and oxidative stress contribute to endothelial dysfunction, organic damage of arterial wall, plaque formation and destabilization with subsequent higher risk of CV events (8, 9). Similarities of chronic inflammatory mechanisms and immune dysfunction processes between CV disease and ARDs further support this hypothesis and, although partially controlled by endogenous anti-inflammatory mechanisms, this chronic, low-grade activation and dysfunction of vascular endothelium is likely to induce and foster accelerated atherosclerosis and CV disease in these patients (8, 9). On the other hand, the contribution of chronic inflammation to precocious atherosclerosis in patients with SSc and SS is less clear, mostly due to the different pathogenesis and systemic features of these diseases. Microvascular disease is a pathognomonic hallmark of SSc and different factors, including dysregulation of vascular tone, defective angiogenesis, endothelial injury/activation elicited by the activation of innate and adaptive immune response and functional defects of progenitor endothelial cells, have been advocated as main pathogenic mechanisms underlying endothelial damage in the disease (10). Similarly, in SS, the pathogenesis of endothelial damage and accelerated atherosclerosis is still unclear (11). Indeed, SS is a chronic immune-mediated inflammatory disease characterized by glandular and systemic manifestations sharing many clinical and autoimmune similarities with RA and SLE (12). However, unlike RA and SLE, the disease is characterized by a low-grade systemic inflammation and by a slow and benign evolution, often not requiring immunosuppressive therapies or high dose corticosteroid treatment (13, 14). SS, therefore, represents an interesting model to analyze the direct effect of autoimmunity and chronic inflammation on atherosclerosis without therapy interference. In this setting, there is evidence that functional and organic subclinical atherosclerosis, characterized by higher prevalence of endothelial dysfunction, aortic stiffness, increased arterial wall intima-media thickness, left ventricular dysfunction and reduced coronary flow reserve, can been detected in young SS patients in comparison to healthy age-matched controls (11). Moreover, there is also evidence that some traditional CV risk factors are more prevalent in these patients in comparison to general population and, more importantly, some of these factors may exert a direct role in determining higher prevalence of subclinical atherosclerosis and higher risk of CV events, as recently observed (15–17). However, the contribution of autoimmune mechanisms and chronic inflammation to subclinical atherosclerosis in SS is still uncertain and studies are ongoing. Thus, this review aims to address the role of inflammation in contributing to increased risk of atherosclerosis in SS patients and the relevance of specific inflammatory biomarkers in the determination of CV risk in these patients.

## Role of Inflammation in the Pathogenesis of SS

Inflammatory infiltration of exocrine glandular parenchyma around the ductal epithelium represents the hallmark of the disease and glandular epithelial cells are key regulators of local immune response. The chronic inflammation observed in SS is the result of a complex and still unexplored altered balance in local and systemic production of specific cytokines, which regulate the heterogeneity and cellular composition of inflammatory infiltrate (14). The glandular infiltrate is usually organized in focal aggregates and is mainly characterized by T and B lymphocytes and, to a lesser extent, by macrophages, plasmacytoid dendritic cells and natural-killer cells (18). Phenotype and severity of inflammatory cell infiltration change according to disease stage and correlate to systemic extra-glandular manifestations suggesting that local inflammatory and immune response is linked to systemic features of the disease (18). Several pathways mediate inflammatory damage in SS pathogenesis.

### Cytokine Involvement

Altered cytokine network and abnormal cytokine production play a pivotal role in the organization and maintenance of glandular infiltrate. Salivary epithelial cells produce several cytokines involved both in innate and adaptive immune response, thus playing a pivotal role in the induction of pathogenic events. In particular, at glandular level, epithelial cells represent the major source of interleukin (IL)-1, IL-6, and tumor necrosis factor (TNF)α. In addition, activated salivary gland epithelial cells secrete IL-7, IL-18, and IL-22 which are strictly involved in adaptive immune response and T cell activation (18). Following adaptive immune system activation, CD4+ T cells produce IL-2, IL-10, and interferon (IFN)γ, thus perpetuating salivary gland inflammation and systemic chronic inflammatory reactions (18). On the other hand, T helper (Th)2-derived cytokines, like IL-4 and IL-5, play a major role in the later stages of the disease, are mainly involved in disease progression and promote local B-cell activation (18). Indeed, B cells are essential in the pathogenesis of the disease and the production of cytokines involved in the homeostasis of B cells, like B-cell activating factor (BAFF), is essential for B-cell proliferation. The result of the production of these pro-inflammatory molecules is chronic salivary gland inflammation, T lymphocyte proliferation and ectopic germinal center formation. In this setting, several mechanisms are involved in the induction of inflammatory cytokine transcription and many of these pathways have been demonstrated to act during preclinical stages of the disease. Among these, experimental models of SS demonstrated that up-regulation of apoptotic pathways and apoptotic genes, as caspase 3 and 8, Fas/Fas ligand complex and TNF apoptosis inducing ligand-receptor (TRAIL-R) 1 and 2, is involved in the dysregulation of immune tolerance and mechanisms of tissue infiltration, thus suggesting a pivotal role of apoptotic mechanism in the induction of inflammatory process leading to glandular damage (19). Moreover, toll-like receptor (TLR) signaling activation has been linked to induction of disease pathogenesis and production of various pro-inflammatory cytokines and adhesion molecules. Interestingly, TLR-7 and−9 ligation results in enhanced secretion of cytokines and chemokines by B cells, including IFNα, IL-8, and IL-15, and secretion of IL-6 and IL-10 is increased upon TLR-9 stimulation (20). Finally, altered expression of IFNα- and IFNγ-regulated genes contribute to the IFN signature which exerts a central role in mediating the link between innate and adaptive response in disease pathogenesis (19).

### Role of Interferon Signature

Several studies demonstrated upregulation in SS patients of genes induced by both type I (mainly INFα and INFβ) and type II (INFγ) IFN signature. In order to identify the predominant IFN signature involved in the induction of disease pathogenesis, both type I and II IFNs and their inducible genes have been investigated in peripheral blood and in salivary gland tissue from primary SS patients with different clinical phenotypes. In this setting, transcriptional analysis demontrated upregulation of type I IFN induced gene expression both in the salivary gland and systemically in peripheral blood mononuclear cells, isolated monocytes, and B cells in SS patients (21). Interestingly, type I IFN signature was associated with higher disease activity, biological markers of activity, and BAFF expression (22). The relevance of activated type I IFN system in SS is further strengthened by the depiction of several INFα-producing cells in salivary gland of SS patients leading to subsequent apoptosis of salivary epithelial cells, exposure of endogenous autoantigens to the immune system, upregulation of BAFF expression, increased B cell survival and differentiation, and ultimately autoantibody production and immunocomplex formation (23). Following this evidence, research focused on the investigation of major sources and factors inducing type I IFN expression in primary SS. Plasmocytoid dendritic cells (pDC)s are potent producers of type I IFNs and secrete high concentration of IFNα/β in response to external triggers or endogenous ligands. Intriguingly, CD123-positive pDCs have been identified in salivary tissue of primary SS patients suggesting that activation of these cells through TLR receptors exerts an essential role in local secretion of type I IFNs and IL-12, which, in turn, promotes type II IFN secretion (24). In this setting, increased expression of IFNγ-inducible cytokines, such as IL-12, by infiltrating inflammatory cells and IL-18 by ductal and acinar cells and by macrophages, has been shown in SS glandular tissue (25). In conclusion, activation of pDCs and glandular epithelial cells by external or endogenous triggers induce the production of type I IFN through TLRs. Type I IFN will lead to BAFF release and activation of B cells with consequent auto-antibody production. Moreover, type I IFN may directly activate T cells which secrete pro-inflammatory cytokines, thus contributing to induce a pro-inflammatory environment. In this scenario, a feedback loop may be postulated between IFN-α production and immune complex formation which will amplify the effect of activated type I IFN system on onset or perpetuation of SS.

### Role of Chemokines

Following the mentioned activation pathways, this complex network of cytokines and chemokines is able to regulate the interplay between immune cells, endothelium and epithelium at glandular level and to perpetuate cellular and humoral autoimmune processes systemically. A variety of chemokines, such as CCL3/MIP-1α, CCL4/MIP-1β, IL-8, and CCL5/RANTES, are produced by activated epithelial cells within the salivary gland and orchestrate recruitment and accumulation of lymphocytes at glandular site. Other chemokines, including CXC ligand (L)12, CXCL13, and CCL11, have been demonstrated to mainly regulate the organization of lymphocytes in germinal center-like structures (18). In particular, CXCL13 is a cytokine directly involved in B cell recruitment to salivary gland, is secreted by follicular dendritic cells and some T cell subsets as follicular Th cells and is recognized as an useful biomarker in SS.

### Role of T Cells

Traditionally, SS was considered a disease mainly regulated by a balance between Th1 and Th 2 cells according to the stage of the disease. However, recent studies demonstrated that the Th17 pathway has an important role in disease pathogenesis. Indeed, infiltrating Th17 cells represent the major source of IL-17, a potent pro-inflammatory cytokine regulated by other cytokines highly expressed in SS glandular tissue, like IL-22 and−23, and able to interact with IL-18 to induce IL-6 and IL-8 from salivary gland cells of SS patients (18). An alternative important source of IL-17 in SS is represented by CD4^−^ CD8^−^ double negative T cells, which are expanded in peripheral blood of SS patients and infiltrate salivary glands (26). Remarkably, IL-17 is highly expressed in salivary glands of SS patients with disease duration <10 years and is mainly detectable in the serum of patients with longer disease duration, suggesting a peculiar role of this pro-inflammatory cytokine in the induction and perpetuation of disease pathogenic pathway (27). Finally, enhanced expression of inflammatory cytokines has been also demonstrated in vessel walls of glandular tissue where they mediate upregulation of adhesion molecules. In particular, IL-1β, IL-4, IFNγ, and TNFα upregulate expression of intercellular adhesion molecule-1 (ICAM-1), vascular cell adhesion molecule-1 (VCAM-1) and E-selectin on endothelium cells, thus reinforcing the important role of inflammatory molecules in recruiting and regulating the glandular infiltrate in SS patients (19).

In conclusion, pro-inflammatory cytokines are key mediators to SS etiopathogenesis by inducing a complex signal network, which regulates the homeostasis of different cell types at glandular level and the immune responses in the local microenvironment of the disease. Moreover, their systemic activity is pivotal to sustaining chronic inflammation and autoimmune damage in extra-glandular tissues and supports the relevant contribution of chronic systemic inflammatory burden to disease pathogenesis and clinical manifestations.

## Role of Inflammation in Atherosclerosis Pathogenesis

**Figure 1:**
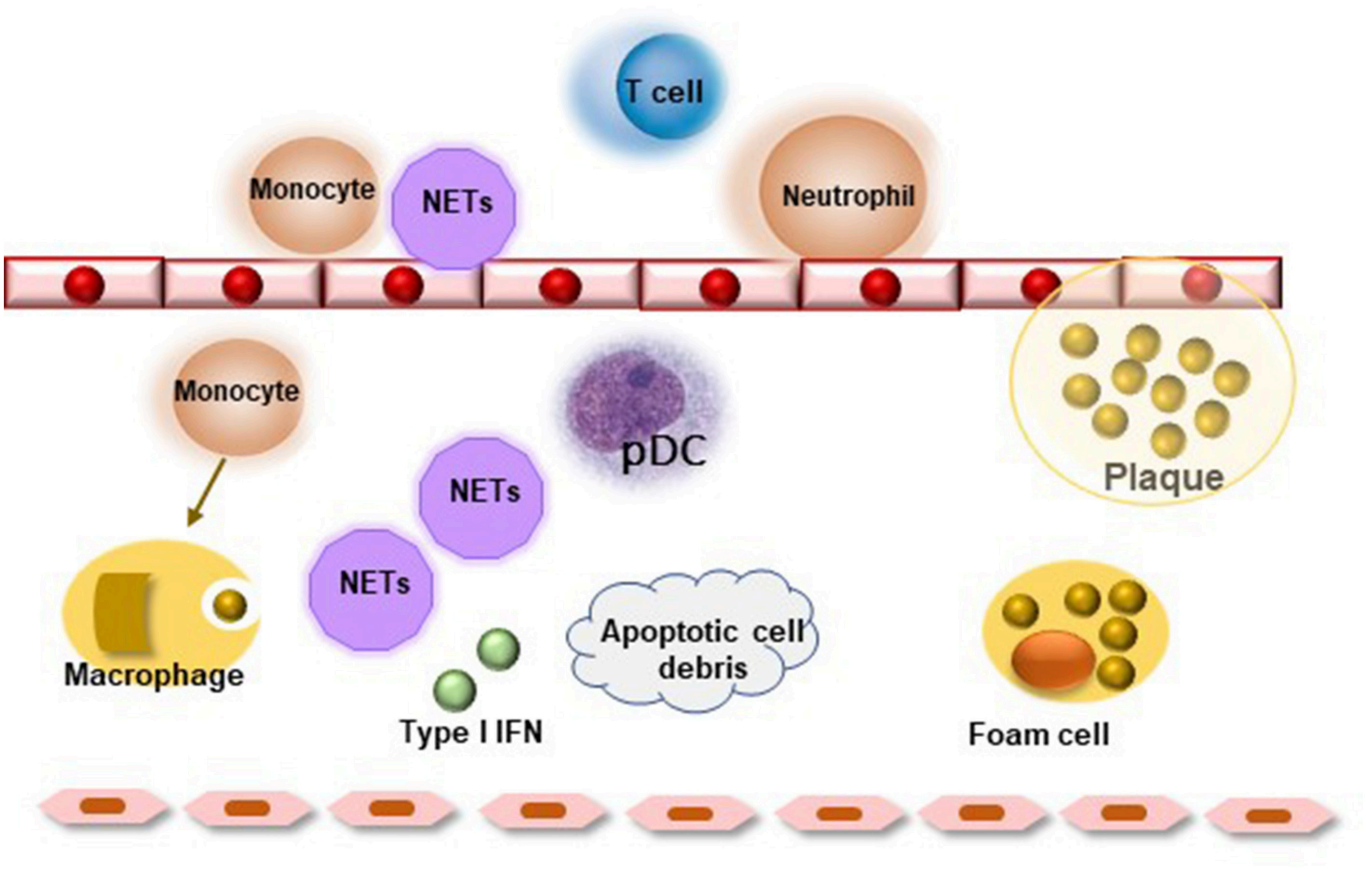
Role of neutrophil extracellular traps (NETs) in atherosclerosis. Neutrophils activate leukocytes, monocytes, and endothelial cells creating a pro-inflammatory milieu resulting in endothelial dysfunction. Lesional NETs and apoptotic cell debris may activate resident plasmocytoid dendritic cells (pDC) favoring type I interferon response, which leads to further activation of lesional leukocytes and release of pro-inflammatory mediators. The inflammatory environment favors plaque destabilization and rupture.

Several *ex-vivo* and *in vitro* studies in the last years explored and analyzed cellular and molecular mechanisms by which chronic inflammation and inflammatory molecules drive atherosclerosis, especially in systemic ARDs (1, 28). In this setting, endothelial cells represent the key players in the initiation of atherosclerotic damage and endothelial activation triggers upregulation of numerous cell adhesion molecules such as ICAM-1, VCAM-1, and selectin, which in turn, mediate interaction and recruitment of inflammatory cells within vessel wall. Pro-inflammatory cytokines, such as monocyte chemoattractant protein-1 (MCP-1) and IL-8, enhance this process by increasing monocyte and macrophage migration under endothelial layer. Subsequently, infiltration of damaged arterial wall by monocyte/macrophage cells promotes the uptake, by macrophage TLRs, of oxidized low-density lipoprotein (ox-LDL), thus leading to lipid accumulation and foam cell formation (1, 28). In this setting, it is has been observed that type I IFNs also contribute to atherosclerotic inflammatory damage, thus highlighting an interesting shared inflammatory mechanism SS and atherosclerosis pathogenesis. Indeed, in mouse models of atherosclerosis, IFNβ enhances endothelial cell-macrophage interaction favoring both leukocyte migration in atherosclerotic site through the induction of CCL5-CCR5 axis and plaque destabilization (29). Moreover, as observed in SS, pDCs, which may be detected in atherosclerotic lesions, represent major source of type I IFNs. Self-apoptotic debris released by necrotic cells or neutrophil-derived proteins, known as neutrophil extracellular traps (NETs), have been demonstrated to stimulate pDCs in the vessel wall inducing type I IFN response and atherogenesis (30, 31). In particular, NETs and material release by apoptotic and necrotic cells, by TLR7/TLR9 binding, induce a strong IFNα response which may prime neutrophils to stimulate NET formation, triggering a positive feedback amplification (31) (Figure 1). Of interest, increased expression of TLRs, which have been implicated in the pathogenesis of ARDs, has been detected on the surface of endothelial cells, thus suggesting an intriguingly link between atherosclerotic endothelial damage and autoimmune disease pathogenesis (9).

Moreover, macrophage activation is associated with release of other inflammatory mediators and reactive oxygen species to amplify inflammatory reaction. It has been demonstrated that oxidative stress has an important role in the atherosclerotic process by promoting the oxidation of LDL and, in this context, there is evidence that ox-LDL proteins are involved in all atherosclerosis steps, starting from recruitment of monocytes within arterial wall to foam cell and plaque formation and, possibly, plaque rupture (32). Finally, immune dysregulation, through the involvement of T lymphocytes, further enhances this process. Th1 cells, in particular, secrete several cytokines, such as IFNγ, IL-2, IL-12, and IL-18 and TNFα, which are known to promote and accelerate atherosclerosis, thus contributing to vascular endothelial damage and plaque progression (1, 28).

In this setting, cytokines are directly involved in the differentiation from monocytes to macrophages in the arterial wall and in driving Th cell polarization toward Th1 and Th2 phenotypes (28). Interestingly, pro-inflammatory cytokines, in particular TNFα, IL-6, and IL-18, and adhesion molecules involved in the atherosclerotic process have been associated with endothelial dysfunction, carotid atherosclerosis, CV morbidity and risk of CV events and mortality in patients with systemic ARDs (9). Recently, the role of Th17 cells in driving atherosclerosis deserved considerable attention. In fact, IL-17 has been demonstrated to exert a pro-atherogenic effect by promoting chemokine-dependent infiltration of monocytes and inflammatory cells within the arterial wall, by upregulating the expression of adhesion molecules, including ICAM-1 and E-selectin, and by induction of pro-inflammatory cytokine, such as TNFα and IL-6, involved in atherosclerotic damage (33). In combination with IFNγ, IL-17 reduces the number of smooth muscle cells, increases collagen production and, therefore, favors fibrosis development, thus leading to plaque instability (33). Finally, Th17 cells can also induce the production of IL-21 and IL-22, pro-inflammatory cytokines involved in macrophage accumulation in the arterial wall as well as T lymphocyte activation.

In addition, pro-inflammatory cytokines may induce atherosclerosis by acting on some traditional CV risk factors. Dyslipidemia is directly linked to systemic inflammation and some cytokines as TNFα and IL-6 have been shown to induce a pro-atherogenic profile and insulin resistance in patients with ARDs (9). Chronic inflammation leads to oxidative changes that alter HDL structure, thus impairing the anti-inflammatory and anti-oxidant effect of HDL proteins (9).

**Figure 2:**
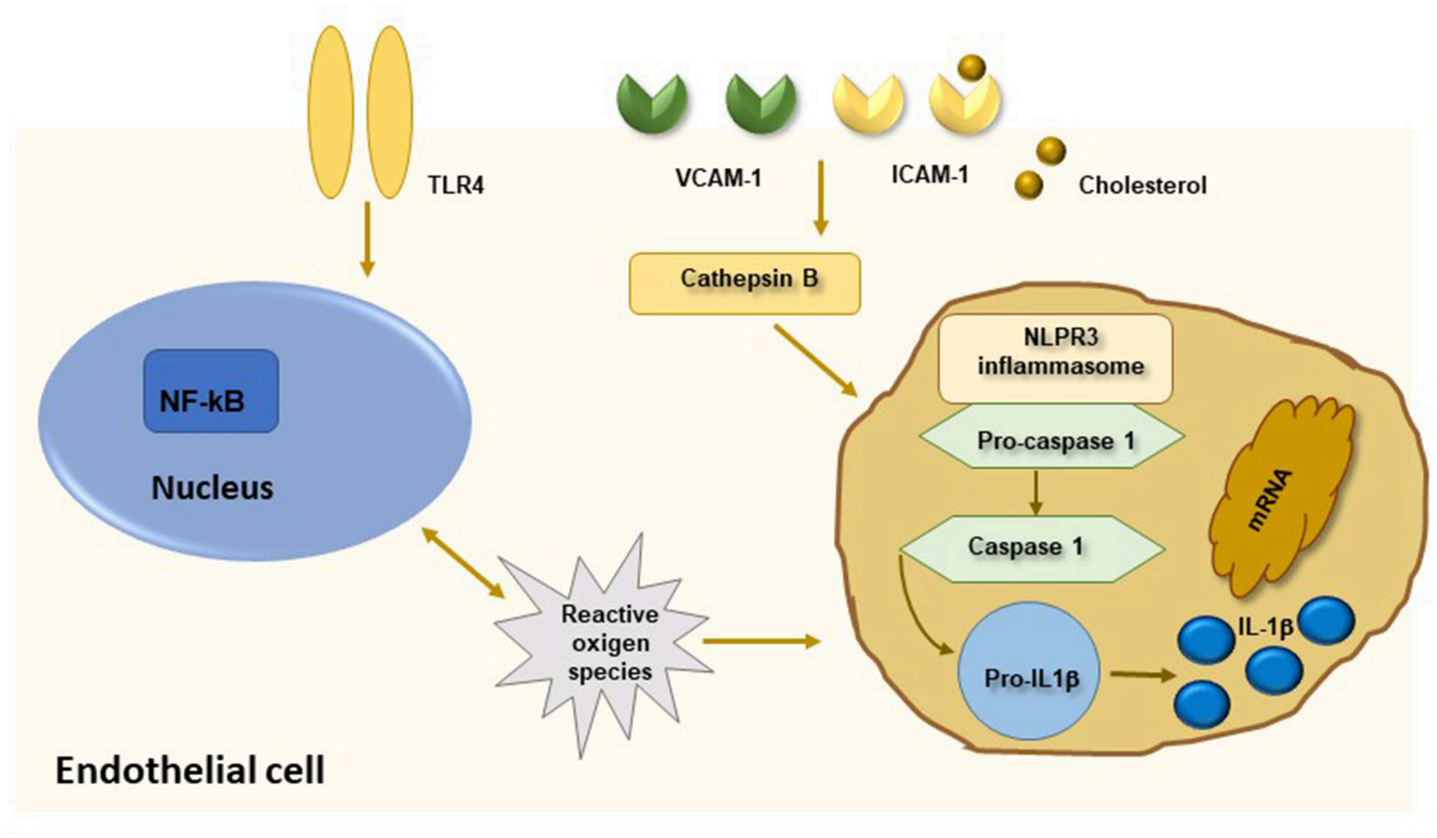
The NLRP3 inflammasome and atherosclerosis. Activation of nuclear factor kB (NF-kB) by endothelial membrane receptors like TLR4 leads to induction of NLRP3 inflammasome. Oligomerization of NLRP3 inflammasome is also mediated by cathepsin B produced by phagocytosis of cholesterol crystals. Activation of NLRP3 inflammasome induces caspase-1 mediated cleavage of pro-interleukin (IL)1β in active IL-1β which mediates a pro-inflammatory state leading to progression of atherosclerosis.

Taken together, these data suggest that pro-inflammatory cytokines and systemic inflammation contribute to all stages of atherosclerosis starting from activation of endothelial layer and recruitment of inflammatory cells within arterial layer to monocyte differentiation and foam cell formation. At later stages of atherosclerosis, these molecules promote apoptosis of arterial smooth muscle cells, matrix degradation and fibrosis with subsequent destabilization and rupture of atherosclerotic plaques. In this intriguing scenario, the evidence that circulating biomarkers of inflammation predict future CV events in patients with ARDs as well as in healthy subjects further reinforce the strict interplay between chronic inflammation and atherosclerosis. Among measurable inflammatory molecules, CRP represents the gold standard biomarker of chronic inflammation with both systemic and local effects and is employed as useful tool in the prediction of CV risk. Several lines of evidence support that CRP, measured by hsCRP, is a strong predictor of future CV events, including stroke, myocardial infarction and sudden death from cardiac causes, in the general population independently of classic CV risk factors (2). Experimental studies demonstrated that CRP is detectable in the vascular intima of atherosclerotic lesions where drives all stages of atherosclerosis (34). In particular, CRP enhances LDL uptake by macrophages and induces foam cell formation. Moreover, CRP contributes directly to thrombosis by inducing the production of pro-coagulant tissue factors with activation of thrombosis at site of inflammation, increases IL-12 production by macrophages, with subsequent induction of IFNγ and ThCD4+ cell differentiation, and triggers the expression and activity of plasminogen activator inhibitor-1, thus leading to atherogenesis through the decrease of fibrinolysis activity. Finally, CRP contribute to plaque neovascularization and instability through increased production of metalloproteinases (34). In this setting, IL-6 represents an adjunctive candidate biomarker for predicting CV morbidity and mortality through its direct induction of CRP hepatic synthesis. Indeed, IL-6 has been linked to atherosclerosis and CV disease in the setting of ARDs as well as in the general population. In particular, IL-6 induces endothelial dysfunction, stimulates the formation of foam cells, reduces the production of HDL-cholesterol and has been associated with cerebrovascular events and peripheral atherosclerotic disease (1). In recent years, interesting studies explored the role of IL-1 signal pathway in the progression of atherosclerosis due to its direct effect on the synthesis of different inflammatory cytokines, including IL-6 and CRP. A direct demonstration of IL-1β, the circulating form of IL-1, as biomarker of atherosclerotic risk is currently lacking due to technical problems in plasma cytokine measurement. However, recent studies demonstrated that IL-1β and IL-18 are activated by caspase-1 in the setting of Nod-like receptor family protein 3 (NLRP3) inflammasome (1, 9). Enhanced expression of NLRP3 inflammasome has been depicted in carotid atherosclerotic plaques and is associated with plaque vulnerability. Moreover, cholesterol stored in macrophages induces IL-1β production by an inflammasome-mediated pathway, thereby suggesting a potential link between cholesterol deposition within arterial wall and macrophage/monocyte promotion of local atherosclerotic progression (Figure 2) (35).

In conclusion, the contribution of inflammation to the pathophysiology of atherosclerosis is complex and not still completely investigated. Undoubtedly, the evidence that inflammatory systemic ARDs, usually characterized by chronically elevated levels of pro-inflammatory cytokines, are associated with an increased risk of CV disease strongly links inflammation to the complex pathogenic pathways contributing to atherosclerotic arterial damage. Moreover, the demonstration that modulation of inflammatory response in patients with systemic ARDs may reduce CV risk and incidence of CV events further supports the inflammatory hypothesis as relevant player, in association with other factors, in the induction and perpetuation of atherosclerotic damage (1). In this setting, multiple pathways have been identified as potential therapeutic targets for the prevention and treatment of CV disease. Recently, the Canakinumab Anti-Inflammatory Thrombosis Outcomes Study (CANTOS) trial demonstrated that administration of canakinumab, an inhibitor of IL-1β activity, is associated with a significant reduction of recurrent CV events in patients with previous myocardial infarction and persistently elevated levels of hsCRP further supporting the hypothesis that controlling chronic inflammation is important for the prevention of recurrent CV events in the general population (1).

## Atherosclerosis in SS: the Contribution of Inflammation

As widely demonstrated in RA and SLE patients, recent evidence now supports that SS patients are characterized, in comparison to general population, by nearly one and a half-fold increased risk of both CVD and cerebrovascular events early during the course of the disease (16, 17). This encouraged to perform a number of investigations aimed to understand mechanisms underpinning this enhanced risk.

First of all, the disease itself emerged as predictor of subclinical atherosclerosis independently by other CV risk factors (36). In addition, increasing evidence suggests that some traditional CV risk factors, namely hypertension, dyslipidaemia and metabolic syndrome, are more prevalent in SS in comparison to age and sex-matched general population and that these factors may exert a role in determining both higher prevalence of subclinical atherosclerotic damage and increased risk of CV events (5, 6, 15). However, how and at what extent these classic CV risk factors interact with disease-specific immune and inflammatory features is still unclear.

### Role of Immunity

Indeed, immune system dysregulation plays a relevant role in the pathogenesis of precocious atherosclerosis in SS patients. Functional impairment of endothelial layer and increased arterial intima-media thickness are associated with circulating anti-SSA/SSB antibodies, two well-established markers of the disease and generally associated with systemic manifestations (37, 38). Moreover, impairment of nitrate-mediated vasodilation and intima-media thickness appear to be associated with, and predictive of, leukopenia, a distinct feature of SS identifying patients with more active disease and higher risk of CV events (16, 37, 38). These data suggest that functional and organic damage of media layer smooth cells in SS may be determined by a cellular infiltration of subendothelial space and the increased serum levels of adhesion molecules, such as ICAM-1 and VCAM-1, detected in SS patients may support that upregulation of these molecules on endothelial layer facilitates leukocyte infiltration within arterial wall with subsequent induction of atherosclerotic damage (38). The evidence that anti-SSB patients had higher frequency of normal aortic stiffness parameters fits well with hypothesis (39). Increased aortic stiffness, in fact, reflects an early and still reversible impairment of large artery function that, as observed in patients with polymyalgia rheumatica can be induced by a rapid increase of inflammatory response rather than by immune system dysregulation (40). In this setting, the demonstration that patients with SSc, a disease not sustained by a systemic high inflammatory burden, have no changes in aortic and upper limb aortic stiffness further supports that inflammation and immune system dysfunction play well distinct roles in determining the different patterns of atherosclerotic wall damage in these patients (41).

### Role of Inflammation

On the other hand, the real contribution of inflammation to atherosclerotic damage in the setting of SS is still unclear. As illustrated in Table 1, some biomarkers of systemic inflammation have been evaluated as predictors of subclinical atherosclerosis or overt CV disease in studies comparing SS patients to normal controls. Two studies found higher CRP levels in SS patients without evidence of significant association with increased risk of atherosclerosis or CV events and higher CRP levels were detected in a Spanish cohort of patients displaying more concomitant classic CV risk factors (44–46). In one study CRP levels correlated with carotid to femoral pulse wave velocity, further supporting the direct link between inflammation and arterial stiffening (47). However, in the majority of studies, CRP or hsCRP levels in patients were not different from those found in normal controls and not associated with subclinical atherosclerosis or CV events, thus supporting the notion that systemic inflammation in SS is usually rather low (36–38, 42–44, 47, 48).

**Table 1:** Inflammatory and endothelial damage biomarkers and atherosclerosis risk in SS.

**Author (Ref.)**	**Patients *N*. (Females)**	Age years^*^	Disease duration (years)^*^	**ATS outcome**	**Biomarkers evaluated**	**Statistically significant results**
Pirildar (42)	25 (96%)	47 ± 10	3 (1–10)^§^	FMD NMV	CRP	None
Vaudo (37)	37 (100%)	48 ± 14	7 (1–15)^§^	c/f IMT Plaque	hsCRP sTM anti-oxLDL anti-Hsp 60/65	sTM higher in patients vs. controls
Rachapalli (43)	25 (96%)	62 ± 9	9 ± 3	ABI	CRP	None
Gerli (38)	45 (100%)	44 ± 8	8 ± 5	FMV NMV	hsCRP sICAM-1 sVCAM-1 Nitrotyrosine	sICAM-1 sVCAM-1 nitrotyrosine higher in patients vs. controls Inverse correlation NMV/sVCAM-1
Perez-De-Lis (44)	312 (95%)	49 ± 2	NR	IHD stroke PAD	CRP	Higher CRP when traditional CV risk factors were ≥3
Juarez (45)	538 (99%)	59 ± 12	NR	MI Stroke	CRP	Higher CRP in patients vs. controls
Atzeni (46)	22 (73%)	60 ± 8	4 ± 1	CFR cIMT§ PWV	CRP Plasma ADMA	Higher CRP and ADMA in patients vs. controls
Balarini (36)	63 (75%)	50 ± 11	9 ± 6	Plaque	hsCRP, IL-1β, IL-6, IL-8, MCP-1, TNFα, and R1/2, Leptin Resistin Adiponectin, Calprotectin	Higher calprotectin, TNF-R2, MCP-1 in patients vs. controls Calprotectin associated with ATS
Demici (47)	75 (100%)	54 ± 9	10 (1–23)§	cfPWV	CRP	cfPWV correlated with CRP
Gravani (48)	64 (96%)	57 ± 12	8 ± 7	c/f IMT Plaque	CRP Fibrinogen DKK1 Sclerostin	Inverse correlation DKK1/plaque
Sabio (39)	44 (100%)	52 (42–44, 47–56)§	6 (3–9)§	cfPWV	CRP Fibrinogen	None

* *mean ± SD, otherwise indicated*.

§ *Median (range)*.

*ABI, ankle brachial index; ADMA, Asymmetric dimethylarginine; Anti-oxLDL, anti-oxidizedLDL antibodies; Anti-Hps60/65, anti-heat shock protein 60/65 antibodies; ATS, atherosclerosis; CFR, coronary flow reserve; cf, carotid femoral; DKK1, Dickkopf-related protein 1; FMV, flow-mediated vasodilation; (hs)CRP, (high-sensitive) C-reactive protein; ICAM-1, intercellular adhesion molecule-1; IHD, ischemic heart disease; IMT, intima media thickness; MCP-1, monocyte chemo attractant protein 1; NMV, nitro-mediated vasodilation; PAD, peripheral artery disease; PWV, pulse wave velocity; sTM, soluble thrombomodulin; VCAM-1, vascular cell adhesion molecule-1*.

Recently, calprotectin emerged as putative marker of atherosclerosis in SS (36). Calprotectin, a complex of S100A8 and S100A9 proteins highly expressed in neutrophil cytoplasm, is an important pro-inflammatory factor of innate immunity acting as endogenous damage-associated molecular pattern molecule via TLR-4 activation. In this context, the activation of TLRs by cell-released molecular debris emerges as interesting process linking inflammatory mechanisms driving SS pathogenesis and atherosclerosis. Recent studies highlighted the central role of self-antigens induced type I IFN release by TLR9 activation in hepatosteatosis, insulin resistance and other components of metabolic syndrome, like obesity (49). Moreover, calprotectin has a pro-inflammatory effect on endothelial cells and promotes inflammation *in vivo*. Of note, in SS patients, increased concentration of S100A8/A9 was identified both at glandular site, where correlated with focus score, and in the circulation, where was associated with an increased production of IL-1β, IL-6, TNFα, IFNγ, IL-10, IL-17A, and IL-22 (50). Calprotectin levels correlate with CRP and recent findings suggest that this molecule may be an important prognostic factor for CV diseases independently of conventional risk factors, thus suggesting that calprotectin may be involved in the pathogenesis of CHD via inflammatory processes (36, 51).

In order to explore adjunctive inflammatory mechanisms underlying atherosclerosis in SS, Gravani et al. analyzed the contribution of the Wingless-type (Wnt) signaling pathway (48), a key regulator of inflammation whose soluble Dickkopf WNT signaling pathway inhibitor 1 (DKK1) has been associated with atherosclerosis. In particular, DKK-1 is overexpressed on endothelial cells and in atherosclerotic plaques where enhances the interaction between platelets and endothelial layer and so driving local inflammation and facilitating plaque destabilization and rupture (52). Moreover, plasma levels of DKK-1 correlate with hs-CRP and are independent predictors of CV events (53). The inverse relationship between circulating DKK-1 levels and plaque formation in SS as well as in diabetic patients supports the direct expression of this molecule within the symptomatic plaque and the DKK-1–driven inflammatory loop could be operating in the atherosclerotic lesion, potentially contributing to atherogenesis and plaque destabilization (48). Finally, the demonstration that the purinergic P2X_7_ receptor (P2X_7_R)-NLRP3 inflammasome complex may be involved in the pathogenesis of SS focal sialadenitis through caspase1-mediated IL-18 release highlights still unexplored mechanisms strictly linking inflammatory pathogenesis of SS glandular infiltrate and inflammatory component of arterial wall atherosclerotic damage (54).

### Endothelial Cell Damage

Despite these interesting data, the direct role of these inflammatory biomarkers in driving atherosclerosis in SS patients has not been proven. In this setting, the evidence of increased levels of biomarkers of endothelial activation and damage in SS may suggest other concomitant mechanisms able to increase CV risk in these patients. Enhanced levels of circulating endothelial microparticles have been detected in a SS cohort, especially in patients with longer disease duration, suggesting a chronic endothelial damage and fragmentation not associated with consequent endothelial repair due to progressive exhaustion of endothelial progenitor cell release (55). Other markers of endothelial activation have been demonstrated to be increased in SS in comparison to normal subjects, such as soluble thrombomodulin (sTM) (37), nitrotyrosine (38), and plasma asymmetric dimethylarginine (ADMA) (46). Thrombomodulin is a membrane glycoprotein with high-affinity receptor activity for thrombin on the endothelial cell surface, implicated in endothelial regulation of fibrinolysis and coagulation. The soluble fragment of the glycoprotein (sTM) reflects the degree of endothelial damage and increased serum level of sTM have been detected in subjects with coronary artery disease, stroke and peripheral occlusive arterial disease (56). Moreover, TM expression in atherosclerotic lesions has been reported to promote atherosclerosis through its mitogenic activity in vascular smooth muscle cells suggesting that sTM has a direct role in atherosclerotic lesions and endothelial damage rather than reflecting an inflammatory process (57). Interestingly, the detection of nitrotyrosine-modified proteins in both plasma and atherosclerotic lesions from patients with coronary artery disease and also from atherosclerotic prone mice suggests that oxidative stress may be a concomitant trigger of atherosclerosis in SS (58). Activation of inflammatory cells into the subendothelial space is tightly associated with generation of reactive oxygen and nitrogen species, which could mediate protein and lipid modifications. Protein nitration is a post-translational modification caused by nitric oxide and interaction between oxidative stress and inflammation promotes plaque formation and rupture (58). Moreover, oxidative stress promotes the formation of ox-LDL proteins which play an important role in different stages of atherosclerosis, as previously illustrated. In this setting, the demonstration that SS patients display higher levels than controls of nitrotyrosine and ADMA, a potent endogenous inhibitor of nitric oxide synthase, suggests that these biomarkers may reflect endothelial dysfunction and damage and enhanced oxidative stress (38, 46). Similarly, the higher levels of soluble adhesion molecules ICAM-1 and VCAM-1 described in a SS cohort may be expression of chronic damage of endothelial layer consequent to subintimal infiltration of inflammatory cells within arterial wall (38).

## Conclusions

The markers of endothelial activation and damage and of chronic inflammation investigated until now failed to result predictors of subclinical atherosclerosis or to be associated with increased risk of CV events in SS patients. This may suggest that other mechanisms are implicated with increased prevalence of subclinical atherosclerosis in SS or that these biomarkers exert a different mechanism in the pathogenesis of endothelial damage and in the induction of atherosclerosis. Surely, the relationship between the disease itself and inflammatory and immune dysfunction factors is quite complex and still to be clarified. Disease activity measured by EULAR SS disease activity index did not correlate with subclinical atherosclerotic damage, probably due to the low disease activity index frequently detected in these patients (39, 48). However, patients with systemic extra-glandular involvement, including central nervous system, appear to be characterized by higher risk of CV events and, on the other hand, patients with more concomitant traditional CV risk factors present higher prevalence of extra-glandular manifestations, in particular liver and central nervous system involvement, in comparison to those without traditional risk factors (16, 44). Actually, chronic damage more than disease activity seems to be associated with higher prevalence of aortic stiffness in SS patients independently by Framingham score (48). As already stated, SS is usually characterized by a benign course with long periods of quiescence. Of consequence, a single measurement of disease activity could not reflect cumulative damage of inflammation. On the other hand, chronic damage may characterize patients with more severe disease over time, often requiring immunosuppressive therapies and higher corticosteroid doses. In this context, it is interesting to note that corticosteroid treatment has been associated with higher risk of CV events in an Italian SS cohort (16).

**Figure 3:**
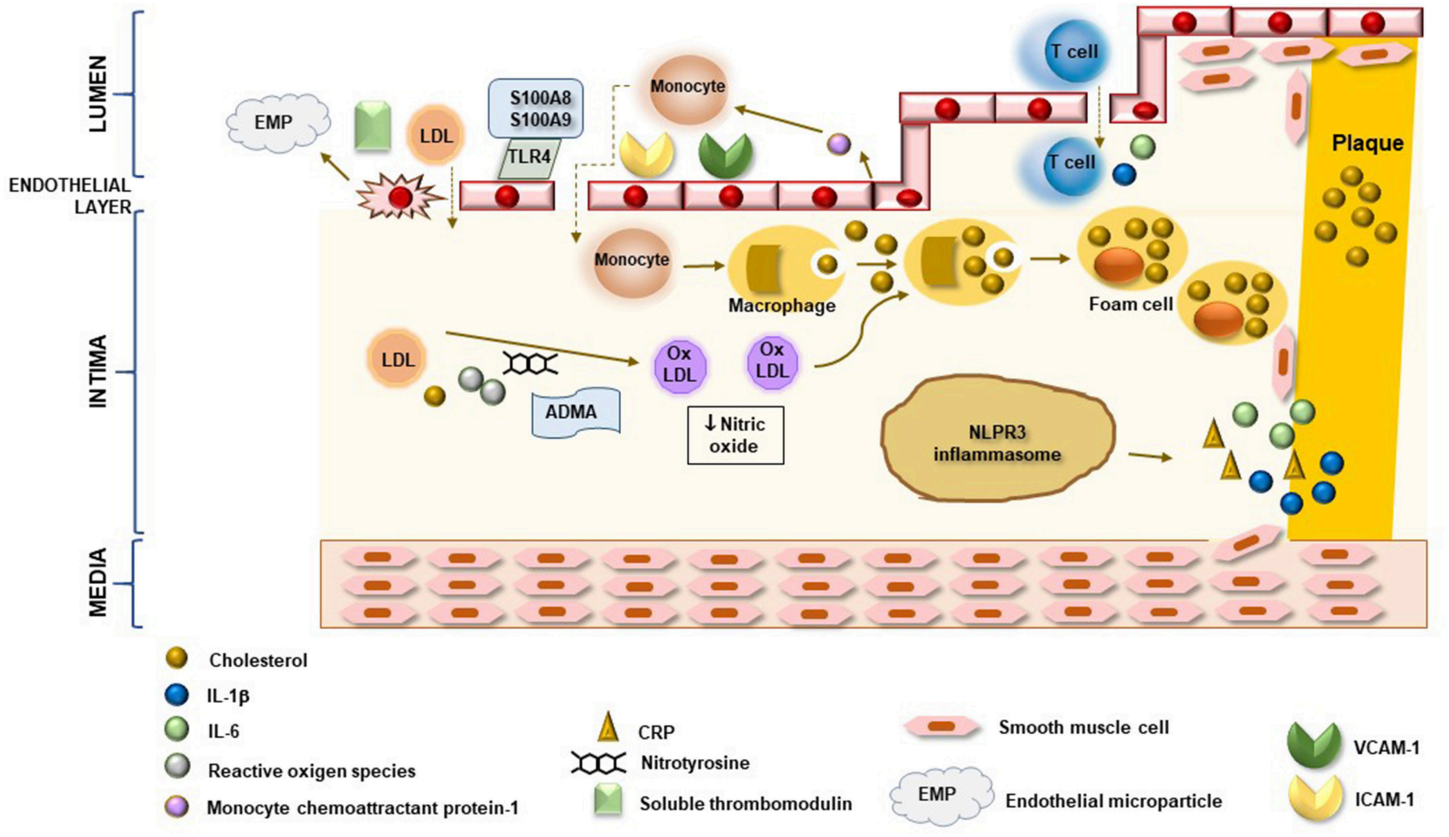
Potential inflammatory mechanisms associated with atherosclerotic arterial wall damage in SS. In SS, both direct damage of endothelial cells and over-expression of adhesion molecules enhances migration of monocytes and lymphocytes in sub-endothelial space as well as low-density lipoprotein (LDL) uptake. Increased production of monocyte chemoattractant protein-1 by endothelial cells and interaction of calprotectin S100A8/S100A9 complex with endothelial layer via TLR4 perpetuate endothelial damage and inflammatory cell infiltration. In vessel intima, reactive oxygen species and oxidative stress mediators, like ADMA and nitrotyrosine, are able to induce LDL oxidation to form ox-LDL. Phagocytosis of cholesterol and ox-LDL by monocyte triggers their differentiation in macrophages and foam cells which are directly involved in plaque formation. Moreover, cholesterol stored in macrophage cells induces caspase 1-mediated pro-IL1β cleavage by NLPR3 inflammasome pathway and release of pro-inflammatory cytokines, including IL-6 and CRP. Chronic endothelial and sub-endothelial space inflammatory damage leads to activation and migration of media smooth muscle cell to intima layer with subsequent formation of fibrous atherosclerotic plaque. Enhanced release of inflammatory molecules by T lymphocytes, in association with all other mechanisms, induces plaque destabilization and rupture.

Taken together, these data suggest that classic CV traditional risk factors and immune system dysregulation exert a central role in the pathogenesis of atherosclerosis in SS patients even though the mechanisms by which they interplay and act need to be further clarified. Indeed, a peculiar poorer immunologic profile, with lower frequency of anti-SSA/SSB antibodies, leukopenia and hypergammaglobulinaemia, seems to identify patients with higher number of traditional CV risk factors, such as hypercholesterolemia (16, 37, 59, 60). On the other hand, a disease phenotype characterized by systemic extra-glandular involvement, presence of organic damage related to chronic active disease and disease- specific immunologic profile should be considered at higher risk to develop CV disease and, of consequence, more aggressively treated to prevent this risk. In this context, research should be addressed to better clarify the contribution of inflammation to atherosclerosis development in SS. As mentioned above, CRP serum concentrations in SS are usually similar to healthy controls and, of more importance, do not appear to be correlated to an increased prevalence of subclinical organic atherosclerotic artery wall damage, except for aortic stiffness. Among other markers of inflammation detected at higher levels in SS patients, only calprotectin has been associated with increased risk of early atherosclerosis, suggesting that future research should be specifically addressed to better investigate the contribution of these molecules to atherosclerotic damage in SS. According to actual knowledge, a possible model of arterial wall atherosclerotic damage in SS may involve the combined action of endothelial layer chronic damage and sub-endothelial infiltration of some inflammatory molecules possibly driving specific pathways within vessel intima (Figure 3).

In conclusion, we believe that addressing interaction between chronic inflammation, immune system dysregulation, CV disease, and other comorbidities in patients with SS has to be acknowledged and directly targeted to improve CV prevention. In the future, better understanding of these specific pathways contributing to atherosclerotic damage in SS patients may lead to the identification and optimization of personalized targeted therapies to be employed in these patients.

## Author Contributions

EB designed and wrote the whole manuscript and prepared the Table. EB, GC, and RG designed the Figure. All Authors revised and approved the manuscript. RG revised and approved the final manuscript draft.

### Conflict of Interest Statement

The authors declare that the research was conducted in the absence of any commercial or financial relationships that could be construed as a potential conflict of interest.
